# Contrasting Green Manure Species and Return Methods Regulate Soil Organic Carbon Mineralization and Retention in Coastal Saline Soil

**DOI:** 10.3390/plants15192913

**Published:** 2026-09-23

**Authors:** Yinjuan Li, Richu Gao, Yuxin Liu, Xuxia Jia, Tongshun Wang

**Affiliations:** 1 College of Hydraulic Science and Engineering, Yangzhou University, Yangzhou 225009, China; 2 Jiangsu Hydraulic Research Institute, Nanjing 210019, China

**Keywords:** green manure, coastal saline soil, SOC mineralization, apparent priming effect, labile carbon fractions, enzyme activities

## Abstract

Green manure application is widely used to improve soil quality in saline-alkaline soils, yet the combined effects of green manure species and return methods on soil organic carbon (SOC) mineralization and field SOC retention remain poorly understood. A field experiment was conducted in coastal saline soil of Jiangsu Province with five treatments: no green manure (CK), alfalfa mulching (AM), alfalfa incorporation (AF), ryegrass mulching (RM), and ryegrass incorporation (RF). A 60-day laboratory incubation was performed to assess SOC mineralization dynamics, apparent priming effects, labile carbon fractions and soil enzyme activities. Our results showed that compared with CK, green manure incorporation significantly increased SOC mineralization and induced positive priming effects. Alfalfa showed higher mineralization rates and stronger priming effects (peaking at day 5, 403.67–551.61%) than ryegrass which peaked at day 21 (395.02–432.50%). After 60 days of incubation, SOC increased by 0.34–0.54 mg·g^−1^ in alfalfa treatments, while it decreased by 1.15–2.42 mg·g^−1^ in ryegrass treatments. In the field, however, SOC content in the 0–10 cm layer was highest in RM (6.88 mg·g^−1^), which was 76.9% higher than that in CK, whereas AF showed the lowest SOC content among the green manure treatments in the 10–20 cm layer. Soil MBC and ROC were highest in AF (196.18 mg·kg^−1^ and 1.973 mg·g^−1^ in the 0–10 cm layer, respectively), while lowest in RM (118.46 mg·kg^−1^ and 1.623 mg·g^−1^). Sucrase and cellulase activities were significantly higher in AF than in other treatments, while RM showed the lowest enzyme activities. These results indicated that alfalfa incorporation stimulated microbial activity and carbon turnover, whereas ryegrass mulching suppressed decomposition and favored carbon retention. Ryegrass mulching is recommended as a promising practice for enhancing short-term SOC retention in this study.

## 1. Introduction

Coastal reclaimed land represents an important reserve of arable land in China and plays a critical role in alleviating land resource shortages and ensuring national food security. However, newly reclaimed coastal soils are commonly characterized by high salinity, low nutrient availability, poor soil structure and low microbial activity, all of which severely constrain crop growth and sustainable agricultural production [1,2]. Salinity stress can alter microbial metabolism and soil enzyme activity, thereby limiting organic matter decomposition, nutrient transformation, and soil carbon cycling [3]. As a key component of soil fertility and ecosystem functioning, SOC contributes to soil structural stability, microbial functioning, and long-term productivity [4,5]. Therefore, increasing SOC while restoring soil biological functions has become a major objective for the sustainable utilization of coastal saline soils.

Green manure application has been widely recognized as an effective measure for improving saline soils and enhancing SOC accumulation [6]. Previous studies have shown that green manure can increase nutrient availability, and contribute to SOC accumulation through residue input and transformation [7]. However, other studies have reported that green manure addition can stimulate SOC decomposition, thereby increasing carbon losses from soil [8,9]. These contrasting findings suggest that the effect of green manure on SOC retention is not determined solely by the amount of carbon returned to soil, but also by the extent to which organic carbon is mineralized following residue addition [10]. This consequently leaves the net effect of green manure on SOC retention uncertain. Therefor, further investigation is needed to clarify how green manure regulates SOC mineralization and carbon retention, particularly in coastal saline soils.

Although labile organic carbon fractions, including dissolved organic carbon (DOC), readily oxidizable carbon (ROC), and microbial biomass carbon (MBC), account for a relatively small proportion of total SOC, they respond rapidly to external organic inputs and management practices [11,12,13]. Therefore, labile organic carbon has been widely used as a sensitive indicator of short- and medium-term changes in SOC turnover. Meanwhile, extracellular enzyme activities provide direct insights into microbial investment in organic matter decomposition and nutrient acquisition, thereby linking residue inputs with carbon cycling processes [14,15,16]. However, current studies have not paid sufficient attention to the mechanisms of organic carbon accumulation and its correlation with soil microbial functions, particularly enzyme activities and labile carbon fractions, in coastal saline soils under green manure management. Elucidating the key factors and pathways of SOC retention can help identify management options for improving soil fertility and ecosystem function in these degraded systems.

Therefore, a field experiment was conducted in coastal saline soil to compare the effects of returning alfalfa and ryegrass residues through soil incorporation or surface mulching. The objectives of this study were to evaluate the effects of green manure species and return methods on (1) soil organic carbon mineralization dynamics, including cumulative mineralization and priming effects; (2) SOC content and labile carbon fractions (DOC, ROC, and MBC); and (3) soil extracellular enzyme activities. This study provides mechanistic insights into the trade-off between carbon transformation and field carbon retention, and offers a scientific basis for green manure management to restore soil functions and enhance carbon retention in degraded coastal saline agroecosystems.

## 2. Results

### 2.1. Dynamics of SOC Mineralization During Incubation

The changes in SOC mineralization rates during the 60-day incubation period are presented in Figure 1. For all treatments, mineralization rates peaked on day 1 with values ranging from 14.20 to 30.92 mg kg^−1^ d^−1^ and then gradually declined with incubation time. The CK treatment reached a plateau after day 21, with rates ranging from 2.66 to 3.72 mg kg^−1^ d^−1^, equivalent to approximately 18.73–26.20% of its peak rate. In contrast, the green manure treatments showed a plateau only after day 52, with rates ranging from 7.11 to 8.14 mg kg^−1^ d^−1^, equivalent to 26.33–34.58% of their respective peak values. Throughout the incubation, the mineralization rates generally followed the order AF > AM > RM > RF > CK, with AF consistently showing the highest rates and CK the lowest.

### 2.2. Cumulative SOC Mineralization and Mineralization Rate

As shown in Figure 2a, the cumulative SOC mineralization exhibited a continuous increasing trend with incubation time across all treatments. During the first 44 days of incubation, cumulative mineralization in all green manure treatments remained consistently higher than that in CK, following the order AF > AM > RM > RF > CK (Figure 2a). By the end of incubation, cumulative mineralization in AM, AF, RM, and RF treatments significantly increased by 167.42%, 183.61%, 172.97%, and 175.93% compared with CK, respectively (Figure 2b). The cumulative mineralization rates at day 60 ranged from 13.36% to 14.17% across green manure treatments, all significantly higher than that of CK (Figure 2c).

### 2.3. SOC Apparent Priming Effects and Changes in SOC After Incubation

The temporal dynamics of *PE* are presented in Figure 3a. All green manure treatments induced positive PE throughout the incubation, with fluctuating intensities. The alfalfa treatments (AM and AF) exhibited similar patterns, peaking at day 5 (403.67–551.61%) and declining thereafter, whereas the ryegrass treatments (RM and RF) peaked later at day 21 (395.02–432.50%). Cumulative *PE* over the 60-day incubation ranged from 2434.57% to 2730.81%, with no significant differences among the four green manure treatments (Figure 3b). Two-way ANOVA confirmed no significant interaction or main effects for cumulative priming effect (*p* > 0.05; Table S1). Changes in SOC before and after the 60-day incubation are presented in Figure 4. After 60 days of incubation, SOC content in the AM and AF treatments increased by 0.34 and 0.54 mg·g^−1^, respectively, relative to pre-incubation levels. In contrast, SOC content in the RM, RF, and CK treatments decreased by 2.42, 1.15, and 1.07 mg·g^−1^, respectively. These results suggest that, under the 60-day incubation conditions, AM and AF promoted SOC accumulation, whereas RM and RF failed to achieve SOC retention.

### 2.4. SOC and Labile Organic Carbon Fractions Under Different Green Manure Treatments

SOC and labile fractions under different treatments are presented in Figure 5. In the 0–10 cm layer, SOC content followed the order RM > AF > AM > RF > CK; in the 10–20 cm layer, the order was RM > AM > RF > AF > CK. Compared with CK, RM increased SOC by 76.9% in the 0–10 cm layer and by 37.1% in the 10–20 cm layer. AF showed the highest MBC and ROC, whereas RM exhibited the lowest MBC and ROC. AF increased MBC by 53.3% over CK, while RM reduced ROC by 37.1%. Overall, AF tended to have higher labile carbon fractions but lower SOC, while RM showed the opposite trend. The interaction between species and return method for SOC was significant only in the surface layer (*p* = 0.001; Table S1).

Proportions of labile organic carbon fractions to SOC under different treatments are shown in Table 1. In the 0–10 cm soil layer, the ROC/SOC and DOC/SOC ratios in all green manure treatments were lower than those in CK, with the reductions in ROC/SOC for the ryegrass treatments (RM and RF) reaching statistical significance (*p* < 0.05). The interaction between species and return method for ROC/SOC was significant in both the 0–10 cm (*p* = 0.004) and 10–20 cm (*p* = 0.004) layers (Table S1), indicating that the effect of green manure species on ROC/SOC depended on the return method. For MBC/SOC, all green manure treatments were lower than CK, except for RF, which was numerically higher but not significantly different from CK. Two-way ANOVA further indicated a significant interaction for MBC/SOC in the 0–10 cm layer (*p* < 0.001; Table S1), implying that the microbial quotient was influenced by the combined effect of species and method. In the 10–20 cm soil layer, AF significantly increased ROC/SOC and MBC/SOC compared with CK, while RM significantly decreased both ratios (*p* < 0.05). In contrast, AM and RF showed no significant differences from CK.

### 2.5. Soil Enzyme Activities Under Different Green Manure Treatments

Soil extracellular enzyme activities under different treatments are presented in Table 2. In the 0–10 cm soil layer, AM showed the highest polyphenol oxidase activity, although it did not differ significantly from CK. In contrast, RF and RM significantly reduced polyphenol oxidase activity compared with CK. For peroxidase, AF and AM exhibited significantly higher activities than the other treatments. Sucrase activity was significantly higher under AF than under all other treatments, whereas AM and RM showed significantly lower values than CK. The interaction between species and return method for sucrase was significant in both layers (*p* < 0.001 for 0–10 cm and *p* = 0.008 for 10–20 cm; Table S1). For cellulase, AF and CK showed the highest activities and were significantly higher than AM, RF and RM. In the 10–20 cm layer, AM still showed the highest polyphenol oxidase activity, while only RF was significantly lower than CK (*p* < 0.05). For peroxidase, AF, RM and RF were significantly higher than CK, whereas AM did not significantly differ from CK. For sucrase, AF remained the highest, while AM was significantly lower than CK, whereas RM was numerically lower than CK but did not differ significantly from it. For cellulase, AF and AM were significantly higher than RF and RM. A significant interaction for cellulase was also detected in the 10–20 cm layer (*p* = 0.003; Table S1).

## 3. Discussion

### 3.1. Green Manure Quality Controls SOC Mineralization and Apparent Priming Effects

In this study, SOC mineralization exhibited a similar temporal pattern across all treatments, characterized by a rapid increase during the first day of incubation, followed by gradual decline and stabilization thereafter (Figure 1). This temporal pattern reflects active microbial decomposition in the early stage of incubation. The input of fresh green manure likely increased the availability of readily decomposable organic fractions (e.g., soluble sugars and free amino acids) in the soil, thereby stimulating microbial metabolic activity and resulting in a rapid increase in CO_2_ emissions within a short period [10,17]. As incubation proceeded, the depletion of these readily available substrates gradually reduced microbial carbon acquisition. Consequently, the remaining residues became relatively enriched in more recalcitrant compounds such as cellulose and lignin [18], which require greater enzymatic investment for decomposition and therefore contribute less to short-term SOC mineralization [19].

Green manure species significantly affected both SOC mineralization and priming effects. In this study, AF produced greater cumulative mineralization and stronger priming effects than RF (Figure 2 and Figure 3). This difference may be attributable to the higher substrate quality of alfalfa residues, which are generally characterized by a lower C/N ratio and lignin content, whereas ryegrass residues typically have a higher C/N ratio and structural carbohydrate content, leading to slower decomposition [20,21,22,23]. The stronger priming effect under AF further suggests that high-quality residues stimulated microbial activity and enhanced the mineralization of native SOC [10,20]. Notably, incorporation treatments (AF and RF) showed higher mineralization intensities than their corresponding mulching treatments (AM and RM). This response was likely due to the closer contact between residues and soil microorganisms following incorporation, which improved substrate accessibility and facilitated microbial decomposition [24].

### 3.2. Contrasting SOC Responses Between Laboratory Incubation and Field Conditions

At maize harvest, SOC content in the 0–20 cm soil layer was consistently higher under all green manure treatments than in CK (Figure 5), suggesting that green manure application enhanced SOC accumulation during the maize growing season [25]. In the 0–10 cm layer, RM performed best, probably because its slower decomposition prolonged carbon residence time in the surface soil. In addition, surface mulching may have improved the surface micro-environment by enhancing moisture retention, buffering temperature fluctuations, and reducing salt accumulation [26], thereby favoring SOC accumulation. For the incorporation treatments (AF and RF), SOC content in the surface layer may also have been supported by carbon inputs from maize rhizodeposition, particularly root exudates released during crop growth [27,28]. Consequently, no significant difference in surface SOC content was observed between AF and RF.

In the 10–20 cm layer, both ryegrass treatments (RM and RF) tended to maintain higher SOC contents than the corresponding alfalfa treatments (AM and AF) (Figure 5). Unlike surface mulching, incorporation delivered green manure residues directly into the 10–20 cm soil layer, where residue-derived carbon inputs and native SOC turnover jointly determined the change in SOC. Notably, alfalfa returned a greater amount of fresh biomass (3.4 t ha^−1^) than ryegrass (2.8 t ha^−1^), yet the ryegrass treatments still had higher SOC contents. This indicates that the amount of carbon returned was likely not the dominant factor determining SOC accumulation. The incubation experiment showed that alfalfa induced the strongest positive priming effect (Figure 3a), suggesting that enhanced mineralization of native SOC may have partly offset the additional carbon introduced by green manure [29,30]. Consequently, despite the greater input of readily decomposable organic matter, alfalfa incorporation did not result in greater SOC content in the subsurface layer. In contrast, the weaker priming effect associated with ryegrass likely reduced native SOC losses, thereby favoring greater SOC retention in the 10–20 cm soil layer. These findings suggest that subsurface SOC accumulation depends not only on the quantity of carbon inputs but also on the balance between residue-derived carbon stabilization and priming-induced native SOC mineralization [31].

### 3.3. Microbial Functional Indicators in Explaining SOC Accumulation

The microbial and biochemical indicators were generally consistent with the SOC mineralization and priming responses observed in the incubation experiment. In particular, in the 10–20 cm layer, the AF treatment exhibited the highest MBC/SOC (5.71%), ROC/SOC (67.59%), as well as the highest sucrase activity among the treatments (Table 1 and Table 2), suggesting greater relative microbial biomass, a larger proportion of labile carbon substrates, and enhanced carbon turnover [13,32]. These characteristics are consistent with the stronger priming effect observed under AF, suggesting that the accelerated utilization of readily available carbon also stimulated the mineralization of native SOC, thereby partly offsetting the additional carbon introduced by green manure. It should be noted that these priming effects are apparent rather than real, meaning the increased CO_2_ efflux may partly reflect accelerated microbial biomass turnover rather than enhanced decomposition of native SOC. Consequently, the higher MBC/SOC ratios observed in AF could partially result from accelerated microbial turnover rather than representing a genuine increase in microbial community size. This distinction remains a limitation of the present study, and future work using isotopic approaches is needed to further differentiate real from apparent priming effects. By contrast, the RM treatment consistently showed lower MBC/SOC and ROC/SOC ratios together with reduced sucrase and cellulase activities, indicating relatively weak microbial activity and slower carbon turnover. Although ryegrass residues decomposed more slowly and generated lower microbial activity during incubation, their relatively slow mineralization may have reduced decomposition-related carbon losses and favored SOC retention under field conditions. Therefore, the higher SOC content observed under RM was likely attributable not only to continuous residue inputs but also to reduced microbial carbon consumption.

Across the 0–20 cm soil layer, RF and RM showed lower polyphenol oxidase activity than CK, with significant reductions observed in the surface layer and in the subsurface layer for RF, indicating that ryegrass residues had a weaker stimulatory effect on this enzyme. In contrast, AM and AF did not significantly differ from CK, suggesting that alfalfa residues maintained polyphenol oxidase activity at a level comparable to CK. This may be associated with the greater input of phenolic compounds during residue decomposition, which could help sustain this enzyme [33]. Peroxidase activity was significantly higher than CK under AF and AM in the 0–10 cm layer, and under AF, RF, and RM in the 10–20 cm layer, whereas the remaining green manure treatments did not differ significantly from CK (Table 2). This overall enhancement indicates that green manure application stimulated microbial investment in oxidative enzymes involved in degrading recalcitrant compounds [34,35]. This is consistent with Hu et al., who reported that both legume and non-legume cover crops significantly increased soil peroxidase activity [36]. Collectively, these microbial and biochemical responses suggest that green manure species influenced SOC content in association with changes in microbial carbon turnover rather than through changing carbon inputs alone.

### 3.4. Implications and Limitations

The contrasting responses between laboratory incubation and field experiments reflect the different temporal and process scales represented by the two approaches. Incubation experiments primarily capture short-term residue decomposition and microbial carbon turnover under controlled conditions, whereas field SOC dynamics represent the integrated outcome of residue turnover, crop-derived carbon inputs, environmental regulation, and SOC stabilization. Therefore, short-term carbon dynamics observed during laboratory incubation cannot be directly extrapolated to field SOC sequestration [10,31]. Accordingly, although alfalfa promoted rapid carbon turnover during incubation, ryegrass mulching resulted in greater SOC retention under field conditions. These findings indicate that rapid residue decomposition does not necessarily maximize field SOC sequestration, which is ultimately governed by the balance between residue-derived carbon inputs, microbial carbon turnover, and priming-induced native SOC losses [10,37]. Under the conditions of the present study, ryegrass mulching showed greater potential for enhancing short-term SOC retention in coastal saline soils. However, these conclusions are limited by the single rotation cycle and the lack of direct quantification of residue-derived carbon stabilization. Long-term field observations are required to verify whether the advantages of ryegrass mulching observed in this study can be sustained under continuous cultivation in coastal saline soils. In addition, while the present study focused on mechanistic aspects of SOC dynamics, future research integrating crop productivity assessments would further strengthen the practical relevance of these findings for agricultural management in coastal saline regions.

## 4. Materials and Methods

### 4.1. Experimental Site

The field experiment was conducted from November 2023 to October 2025 at the Coastal Research Base of the Jiangsu Hydraulic Research Institute, Dongtai City, Jiangsu Province, China (120°53′ E, 32°51′ N). The experiment covered two consecutive green manure-maize rotation cycles, and all data reported in this study were collected during the second cycle (2024–2025). The site is located on a coastal alluvial plain in the Yangtze River Delta and represents a typical reclaimed tidal-flat agricultural soil. The region has a monsoon climate with an average annual mean temperature of 14.9 °C, mean annual precipitation of 986.0 mm and annual evaporation of 1066.6 mm, respectively. The soil texture was classified as silt loam [38]. The soil had an initial pH of 7.9, SOC content of 3.28 g kg^−1^, bulk density of 1.36 g cm^−3^, electrical conductivity of 154.2–202 μS cm^−1^, and salt content of 3–5 g kg^−1^. Moreover, the initial available phosphorus, available potassium, and available nitrogen contents were 8.8, 114.6, and 110.7 mg kg^−1^, respectively.

### 4.2. Field Experimental Design

A completely randomized design was used with five treatments and three replicates, resulting in a total of 15 plots. The treatments included no green manure during the winter season (CK), ryegrass full incorporation (RF), ryegrass mulching (RM), alfalfa full incorporation (AF), and alfalfa mulching (AM). Each plot measured 8 m × 4 m, and adjacent plots were separated by 50 cm buffer strips to prevent the lateral exchange of water and nutrients.

During the winter green manure season, ryegrass (*Lolium perenne* L.) and alfalfa (*Medicago sativa* L.) were sown in November in rows spaced 30 cm apart. Both species were sown at the locally recommended rate of 45 kg ha^−1^. No chemical fertilizer was applied during the green manure growing period. Before maize sowing, the aboveground fresh biomass of green manure was manually harvested from each plot, weighed, chopped into small pieces, and returned to the corresponding plot. The fresh biomass input was equivalent to 3.4 t ha^−1^ for alfalfa and 2.8 t ha^−1^ for ryegrass. Prior to maize sowing, all plots received a single mechanical tillage operation to a depth of 0–20 cm. In the incorporation treatments, chopped green manure was evenly distributed on the soil surface and then mixed into the topsoil during tillage. In the mulching treatments, tillage was carried out first, after which the same chopped green manure was evenly spread on the soil surface without further mixing. In the CK treatment, tillage was performed but no green manure was applied.

Summer maize (*Zea mays* L., cv. Yufeng 303) was sown on June 25 and harvested on October 8 in 2025. Maize was planted at a row spacing of 55 cm and an in-row plant spacing of 25 cm. The hill-drop method was used, with three seeds per hill and a seeding rate of 25 kg ha^−1^. After emergence, seedlings were thinned to one plant per hill. Compound fertilizer (N:P:K = 29:5:6) was applied as basal fertilizer at a rate of 833 kg ha^−1^ (product mass) before maize sowing, and urea (46% N) was top-dressed at the jointing stage at a rate of 833 kg ha^−1^ (product mass). No irrigation was applied during the experimental period. Weed, pest, and disease control were conducted according to local conventional management practices.

### 4.3. Soil Sampling and Soil Analysis

Prior to green manure sowing in 2024, soil samples were collected from the 0–20 cm soil layer. Fresh samples were manually removed of visible plant residues and stones, placed in insulated coolers with ice packs, and transported to the laboratory. The samples were then air-dried at room temperature, ground, and passed through a 2 mm sieve before use in the indoor incubation experiment.

After maize harvest in October 2025, soil samples were collected from the 0–10 cm and 10–20 cm layers in each plot using a stainless steel auger (4 cm inner diameter) following an “S”-shaped five-point sampling method. For each soil layer, five soil cores from each plot were thoroughly mixed to form one composite sample. The fresh soil was manually freed from debris and divided into two subsamples: one was air-dried at room temperature, ground, and sieved for the determination of SOC and readily oxidizable carbon (ROC); the other was kept refrigerated at 4 °C for the analysis of microbial biomass carbon (MBC), dissolved organic carbon (DOC), and soil extracellular enzyme activities.

### 4.4. Laboratory Incubation Experiment

A 60-day laboratory incubation experiment was conducted with five treatments corresponding to those in the field experiment (AM, AF, RM, RF, and CK), each with three replicates. For each treatment, 100 g of the sieved air-dried soil was placed in 750-mL Mason jars and adjusted to 60% of field capacity. Field capacity was determined on undisturbed soil cores by the cutting-ring method: intact cores were saturated for 24 h, drained on dry sand until gravitational water was removed, and oven-dried at 105 °C to calculate the gravimetric water content. The measured field capacity was 0.31 g g^−1^. At the beginning of the incubation test, pre-incubation for 7 days at 25 ± 1 °C was carried out to restore soil microbial activity. Then air-dried green manure (alfalfa or ryegrass) was ground and added at 1% of soil dry weight. For mulching treatments, the green manure material was placed on the soil surface; for incorporation treatments, it was thoroughly mixed with the soil. Three blank jars (containing only NaOH traps) were used as controls to correct for background CO_2_ absorption during incubation. CO_2_ released during incubation was trapped in 20 mL of 0.5 mol L^−1^ NaOH solution suspended inside each jar. The jars were sealed and incubated at 25 °C for 60 days. On days 1, 2, 3, 5, 8, 12, 16, 21, 26, 31, 36, 44, 52, and 60, the NaOH traps were collected, and the trapped CO_2_ was determined by titration with 0.2 mol L^−1^ HCl after adding BaCl_2_. After each sampling, the NaOH traps were replaced, and all jars were aerated for 30 min. The soil moisture was adjusted using a weighing method to maintain it at 60% of the field capacity. Pre-incubation SOC was measured on air-dried, 0.15 mm-sieved soil before green manure addition. At day 60, visible undecomposed green manure residues were removed by passing the soil through a 0.15 mm sieve and hand-picking, and the remaining soil was homogenized for SOC determination.

### 4.5. Soil Analysis and Calculations

SOC was determined using the K_2_Cr_2_O_7_–H_2_SO_4_ oxidation method with external heating [39]. Soil microbial biomass carbon (MBC) was measured by the chloroform fumigation–K_2_SO_4_ extraction method [40]. Readily oxidizable organic carbon (ROC) was determined using the KMnO_4_ oxidation method, while dissolved organic carbon (DOC) was extracted with deionized water [33,41]. Soil cellulase and sucrase activities were measured using the 3, 5-dinitrosalicylic acid colorimetric method; soil peroxidase and polyphenol oxidase activities were determined using the 1% pyrogallol colorimetric method [42]. In this study, total CO_2_ released during incubation was used as the operational measure of SOC mineralization, which includes CO_2_ derived from both the added green manure residues and the native SOC. The measured values represent total CO_2_ release, and the calculated priming effect represents an apparent priming effect.

SOC mineralization rate (mg kg^−1^ d^−1^) and the cumulative SOC mineralization rate (%) were calculated as follows:

(1) $$\mathrm{SOC}\;\mathrm{mineralization}\;\mathrm{rate}\;(\mathrm{mg}\;{\mathrm{kg}}^{-1}\;d^{-1})\;=\;\frac{C_{i}}{d_{i}}$$

(2) $$\mathrm{Cumulative}\;\mathrm{SOC}\;\mathrm{mineralization}\;\mathrm{rate}\;(\%)=\;\frac{C_{60}}{{SOC}_{0}}\times \;100\%$$

where for each sampling day i, C_i_ is the amount of CO_2_ released up to day i ($\mathrm{mg}\;{\mathrm{kg}}^{-1}$), d_i_ is the incubation time at the i-th sampling (d), C_60_ is the cumulative amount of CO_2_ released during the 60-day incubation period ($\mathrm{mg}\;{\mathrm{kg}}^{-1}$), and the SOC_0_ is the soil organic carbon content measured before the incubation (mg kg^−1^).

The apparent priming effect (*PE*) was calculated as follows [43]:

(3) $$PE\;=\;\frac{Q_{1}\;-Q_{2}}{Q_{2}}\times 100\%$$

where PE is the apparent priming effect, *Q*_1_ and *Q*_2_ are the cumulative total CO_2_ released in the green manure-amended treatment and CK treatment (soil without green manure), respectively (mg·kg^−1^).

(4) $$Cululative\;PE=\sum_{i=1}^{14}{PE}_{i}$$

where *PE_i_* is the apparent priming effect at the *i*-th sampling time point, and 14 is the total number of sampling time points.

### 4.6. Statistical Analysis

Data were recorded and processed using Microsoft Excel 2021. One-way analysis of variance (ANOVA) followed by Duncan’s multiple range test (*p* < 0.05) was performed with IBM SPSS 26 to test the significance of differences among treatments for each indicator. For the four green manure treatments (AM, AF, RM, RF), data were further analyzed using two-way analysis of variance (ANOVA) with green manure species (alfalfa vs. ryegrass) and return method (mulching vs. incorporation) as fixed factors. Time-course data (mineralization rate and apparent priming effect over the 60-day incubation) were presented descriptively to illustrate temporal trends; no repeated-measures ANOVA or treatment × time analysis was performed, and no inferential statistics were applied to these repeated measures. Accordingly, all inferential analyses were restricted to independent endpoint variables, including cumulative CO_2_ release at day 60, cumulative apparent priming effect, and changes in SOC content after incubation. All bar charts and line graphs were generated using Origin 2019.

## 5. Conclusions

In the present study, green manure species and return methods influenced SOC mineralization and carbon retention in coastal saline soil, with the effects varying among the measured endpoints. All green manure treatments stimulated SOC mineralization and induced positive priming effects, with alfalfa producing an earlier priming peak and generally higher mineralization rates than ryegrass, particularly under incorporation. Cumulative priming effects did not differ significantly among green manure treatments. Alfalfa treatments resulted in an increase in SOC content after the short-term incubation, whereas ryegrass treatments showed a decrease. Under field conditions, however, RM produced the highest SOC content in both soil layers and increased topsoil SOC by 76.9% compared with CK. AF showed higher MBC and ROC contents and greater activities of several enzymes involved in carbon decomposition. In contrast, RM resulted in greater SOC content but lower MBC and ROC contents, as well as lower ratios of labile carbon fractions to SOC. Overall, greater short-term carbon mineralization and labile carbon availability did not necessarily translate into greater field SOC retention in this study. Therefore, within the treatments examined, RM showed short-term potential for enhancing SOC retention in the topsoil of the studied coastal saline soil. However, these results are based on SOC concentration rather than SOC stock and on a single reported rotation cycle. Long-term field studies are needed to verify whether these benefits can be sustained.

## Figures and Tables

**Figure 1:**
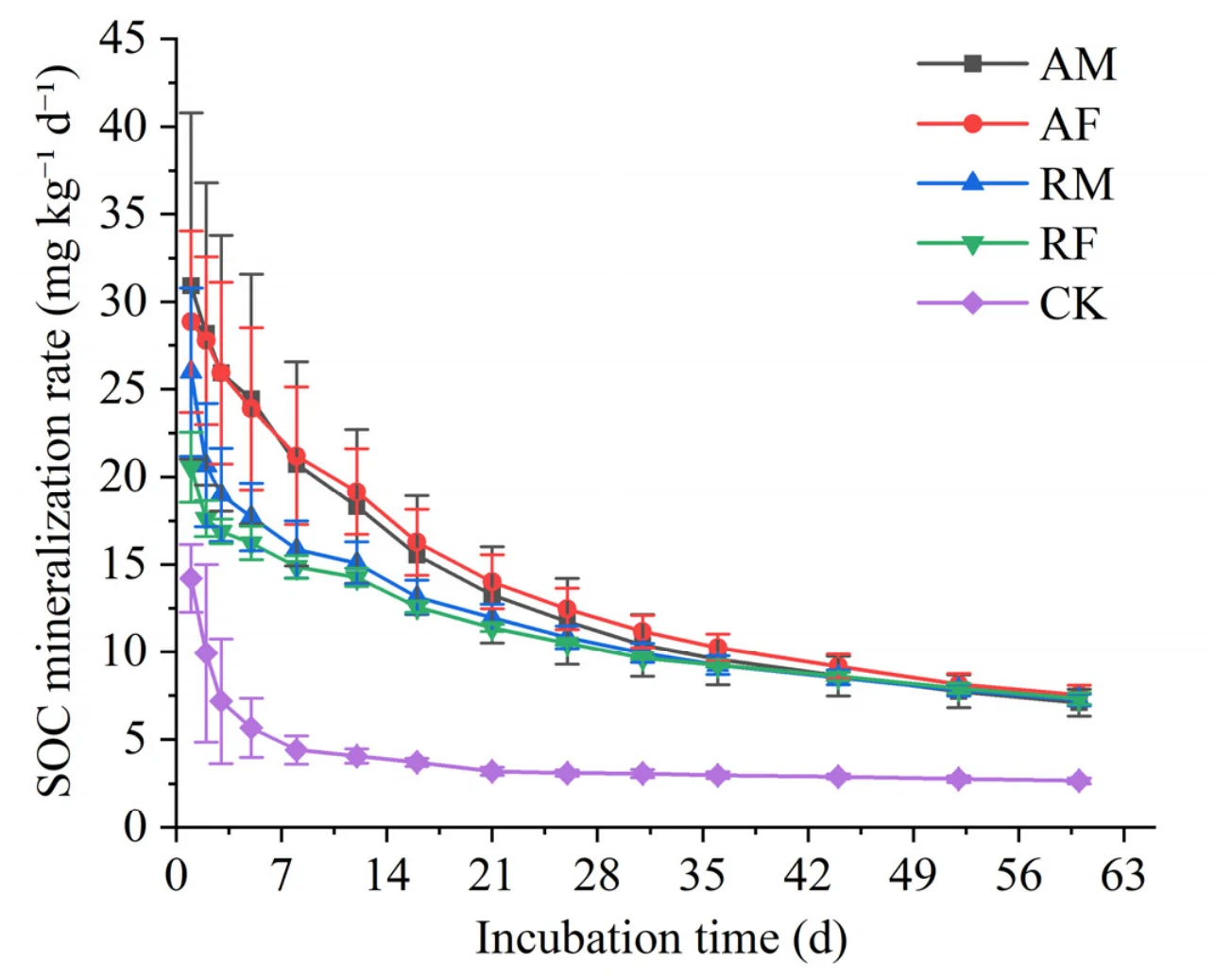
SOC mineralization rates under different treatments over the incubation period.

**Figure 2:**
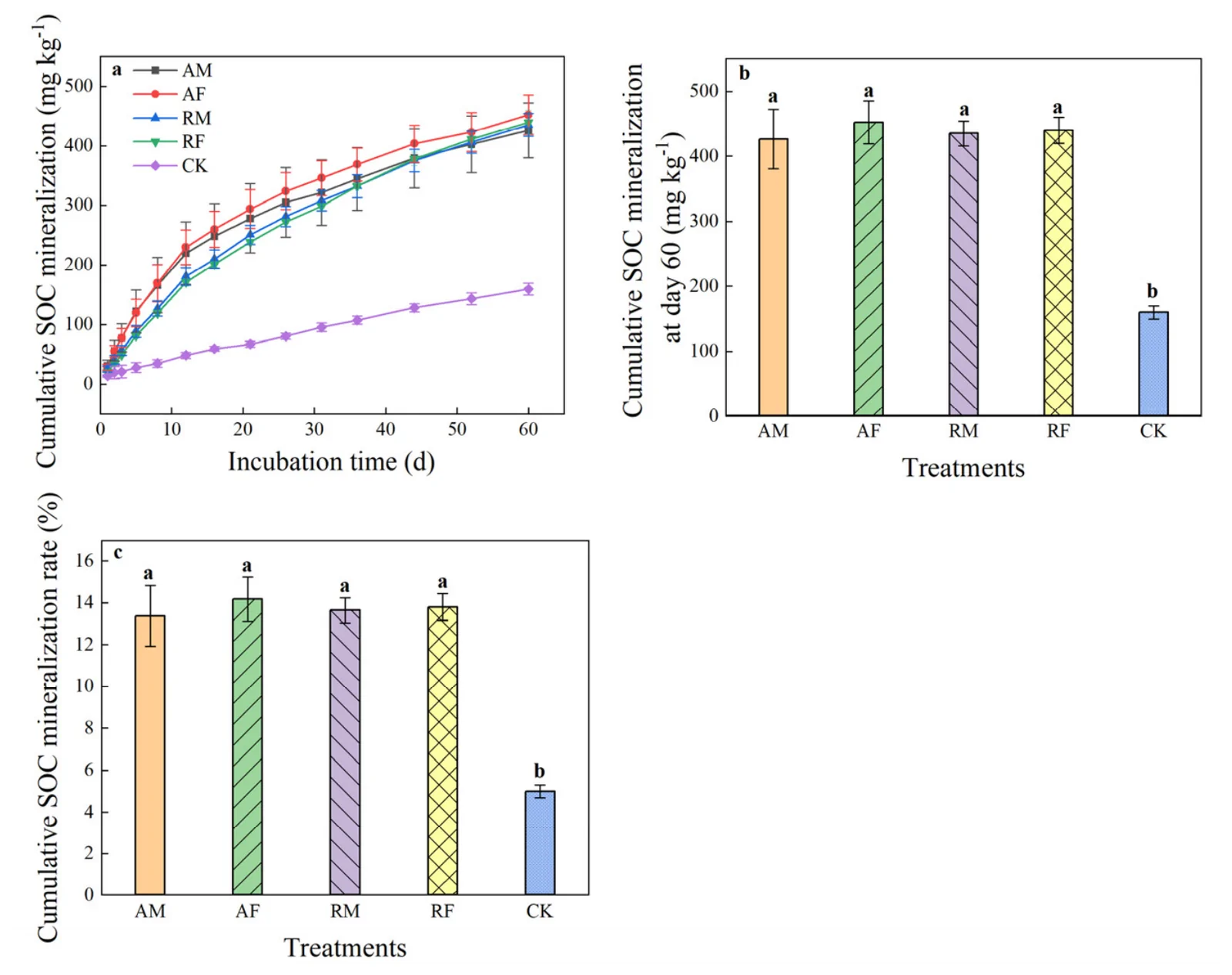
Dynamics of cumulative SOC mineralization (**a**), cumulative SOC mineralized at day 60 (**b**), and cumulative mineralization rate expressed as a percentage of initial SOC (**c**) under different treatments during the 60-day incubation period. Different letters indicate significant differences among treatments at *p* < 0.05.

**Figure 3:**
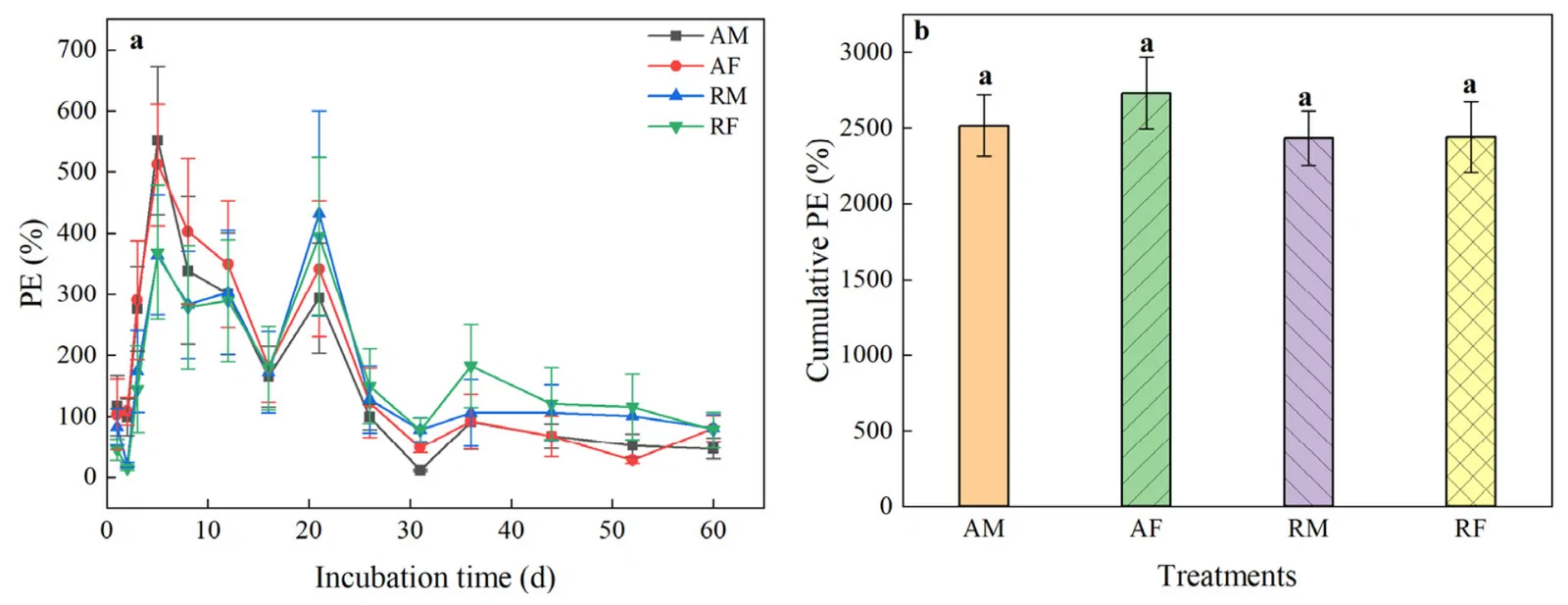
Apparent priming effects during soil incubation (**a**) and cumulative priming effects (**b**) under different treatments over the 60-day incubation period. Different letters above bars indicate significant differences among treatments at *p* < 0.05.

**Figure 4:**
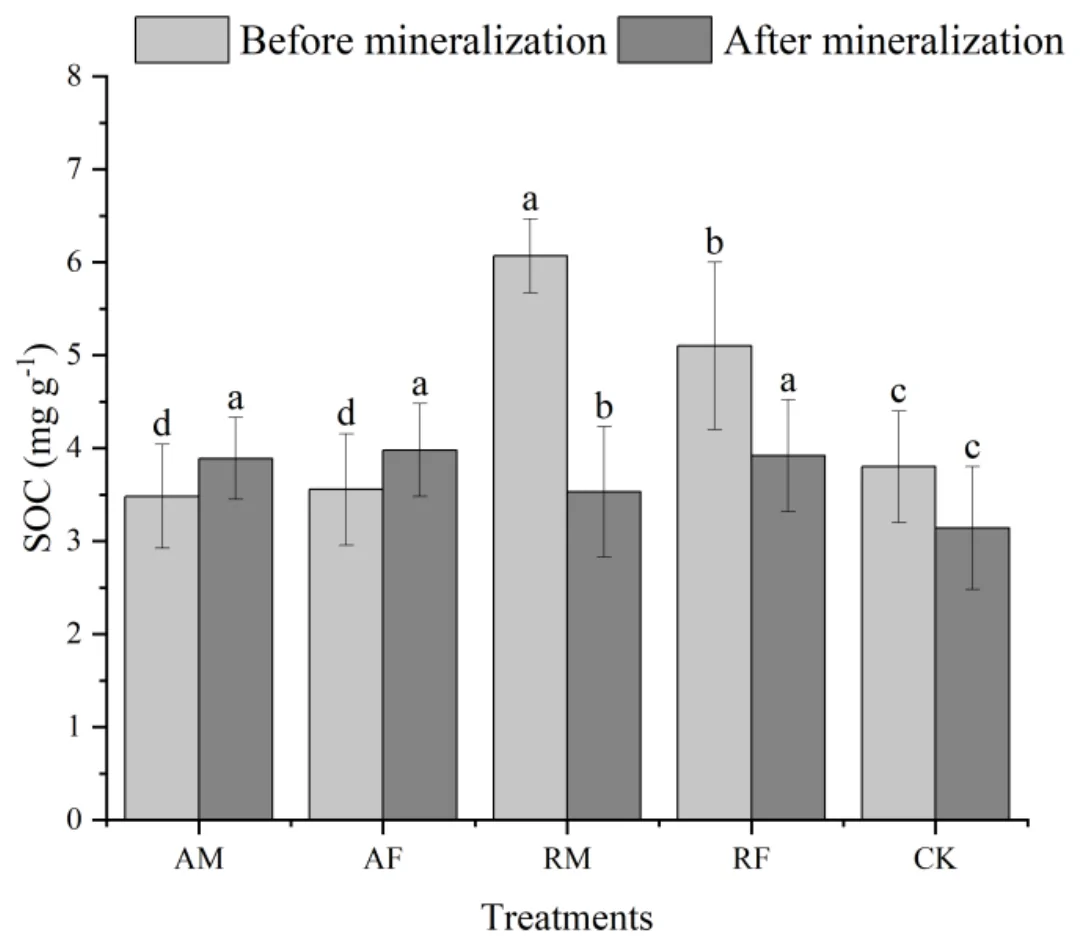
Changes in SOC content before and after the 60-day incubation under different treatments. Different letters above bars indicate significant differences among treatments at *p* < 0.05.

**Figure 5:**
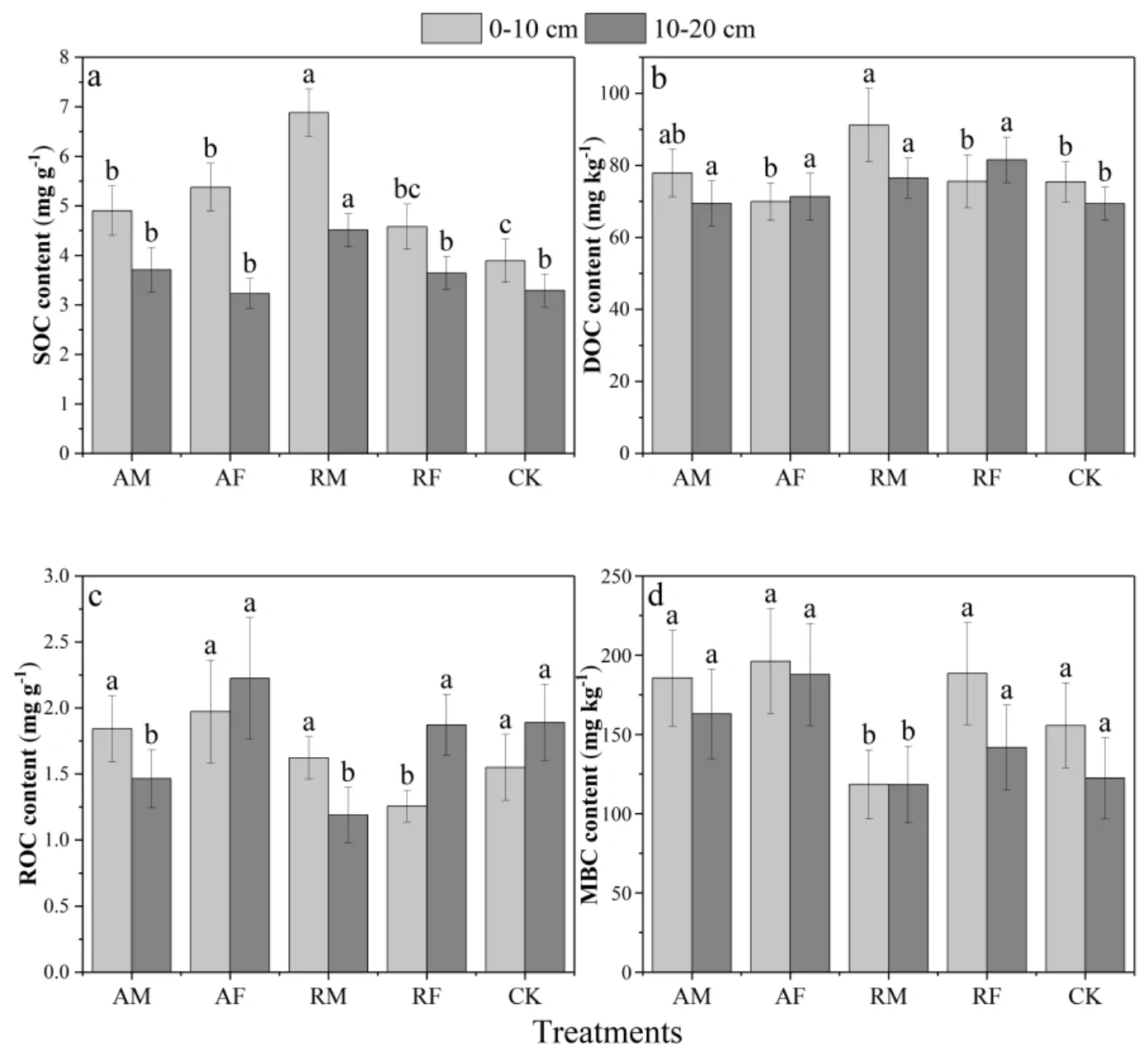
SOC and labile fractions under different treatments (DOC, ROC, and MBC) under different green manure treatments at maize harvest. Different lowercase letters within the same soil layer indicate significant differences among treatments at *p* < 0.05. (**a**) SOC content; (**b**) DOC content; (**c**) ROC content; (**d**) MBC content.

**Table 1:** Proportions of different labile organic carbon fractions in total organic carbon under different treatments.

Soil Depth	Treatment	ROC/SOC (%)	DOC/SOC (%)	MBC/SOC (%)
0–10 cm	CK	39.84 ± 2.16 a	1.94 ± 0.21 a	4.00 ± 0.18 a
	AF	36.74 ± 4.67 a	1.30 ± 0.24 b	3.65 ± 0.17 b
	AM	37.61 ± 2.45 a	1.59 ± 0.19 ab	3.79 ± 0.17 ab
	RF	27.46 ± 1.49 b	1.41 ± 0.21 b	4.11 ± 0.17 a
	RM	23.60 ± 1.28 b	1.32 ± 0.25 b	1.72 ± 0.17 c
10–20 cm	CK	58.54 ± 3.42 b	2.15 ± 0.14 a	3.79 ± 0.37 b
	AF	67.59 ± 7.30 a	1.92 ± 0.15 ab	5.71 ± 0.76 a
	AM	39.46 ± 3.40 c	1.87 ± 0.14 ab	4.39 ± 0.41 b
	RF	51.46 ± 3.34 b	2.10 ± 0.15 a	3.89 ± 0.42 b
	RM	26.36 ± 2.76 d	1.81 ± 0.15 b	2.62 ± 0.35 c

Notes: Data are presented as means ± standard deviation (n = 3). Different lowercase letters within the same soil layer indicate significant differences among treatments at *p* < 0.05.

**Table 2:** Effects of different treatments on soil extracellular enzyme activities.

Soil Depth	Treatment	Polyphenol Oxidase (mg/g/2 h)	Peroxidase (mg/g/2 h)	Sucrase (mg/g/24 h)	Cellulase (mg/g/72 h)
	AF	0.71 ± 0.06 bc	3.23 ± 0.18 a	5.40 ± 0.64 a	0.54 ± 0.04 a
	AM	0.86 ± 0.08 a	3.41 ± 0.17 a	1.20 ± 0.07 c	0.45 ± 0.03 b
0–10 cm	RF	0.66 ± 0.04 c	2.23 ± 0.10 c	3.68 ± 0.37 b	0.40 ± 0.02 c
	RM	0.62 ± 0.03 c	2.65 ± 0.11 b	1.80 ± 0.17 c	0.32 ± 0.01 d
	CK	0.79 ± 0.05 ab	2.57 ± 0.15 b	3.01 ± 0.35 b	0.54 ± 0.03 a
	AF	0.70 ± 0.05 bc	3.28 ± 0.14 a	4.42 ± 0.56 a	0.55 ± 0.06 a
	AM	0.82 ± 0.06 a	2.87 ± 0.11 bc	1.07 ± 0.24 d	0.47 ± 0.04 a
10–20 cm	RF	0.62 ± 0.03 c	2.93 ± 0.18 b	3.59 ± 0.46 b	0.31 ± 0.02 b
	RM	0.74 ± 0.04 ab	3.10 ± 0.16 ab	1.93 ± 0.32 c	0.36 ± 0.03 b
	CK	0.78 ± 0.06 ab	2.65 ± 0.12 c	2.43 ± 0.21 c	0.56 ± 0.08 a

Notes: Data are presented as means ± standard deviation (n = 3). Different lowercase letters within the same soil layer indicate significant differences among treatments at *p* < 0.05.

## Data Availability

The original contributions presented in this study are included in the article/Supplementary Material. Further inquiries can be directed to the corresponding author.

## Supplementary Materials

The following supporting information can be downloaded at: https://www.mdpi.com/article/10.3390/plants15192913/s1, Figure S1. Schematic timeline of the field experiment showing green manure cultivation, residue incorporation, maize growing period, and soil sampling events. Arrows indicate key events; Table S1. Two-way ANOVA results (*p*-values) for soil properties, enzyme activities, and mineralization parameters as affected by green manure species, return method, and their interaction.
