# Supplementary material for: Contrasting Green Manure Species and Return Methods Regulate Soil Organic Carbon Mineralization and Retention in Coastal Saline Soil
Source: Plants (Basel). 2026 Sep 23;15(19):2913. doi: 10.3390/plants15192913 (PMC13667276; doi:10.3390/plants15192913)
Supplement: Supplementary file 1 [file plants-15-02913-s001.zip › plants-4570264-supplementary.pdf]

Supplementary material

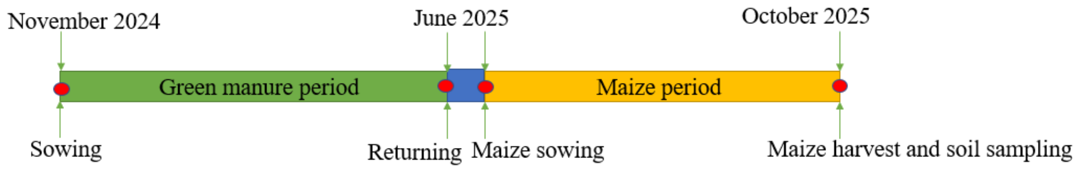

Figure S1 Schematic timeline of the field experiment showing green manure cultivation, residue incorporation, maize growing period, and soil sampling events. Arrows indicate key events.

Table S1. Two-way ANOVA results (*p*-values) for soil properties, enzyme activities, and [illegible]n.

Soil depth	Variable	Species	Returning method	Species × Returning method
0-10 cm	Polyphenol oxidase	ns	0.010	0.018
	Peroxidase	<0.001	ns	ns
	Sucrase	<0.001	<0.001	<0.001
	Cellulase	<0.001	0.003	ns
	SOC	ns	ns	0.001
	DOC	ns	ns	ns
	ROC	0.008	ns	ns
	MBC	ns	ns	ns
	ROC/SOC (%)	0.003	ns	ns
	DOC/SOC (%)	<0.001	<0.001	<0.001
	MBC/SOC (%)	ns	ns	<0.001
10-20 cm	Polyphenol oxidase	ns	0.014	ns
	Peroxidase	0.022	0.010	0.010
	Sucrase	0.039	<0.001	0.008
	Cellulase	<0.001	0.006	0.003
	SOC	ns	ns	ns
	DOC	ns	ns	ns
	ROC	0.003	0.015	ns
	MBC	ns	ns	ns
	ROC/SOC (%)	<0.001	<0.001	0.004
	DOC/SOC (%)	ns	0.004	ns
	MBC/SOC (%)	0.002	0.010	ns
	Cumulative SOC mineralization (day 60)	ns	ns	ns

Cumulative SOC mineralization rate	ns	ns	ns

Note: Values are *p*-values from two-way ANOVA. ns indicates not significant ($p \geq 0.05$). SOC: soil organic carbon; DOC: dissolved organic carbon; ROC: readily oxidizable carbon; MBC: microbial biomass carbon.
